# Unscheduled Return Visits to the Emergency Department after Cardiac Electronic Devices Implantation

**DOI:** 10.5935/abc.20190063

**Published:** 2019-05

**Authors:** Roberto Costa

**Affiliations:** Instituto do Coração (InCor) do Hospital das Clínicas da Faculdade de Medicina da Universidade de São Paulo (HC-FMUSP), São Paulo, SP - Brazil

**Keywords:** Pacemaker, Artificial/utilization, Intraoperative Complications/mortality, Defibrillators, Implantable, Heart Failure, Cardiac Pacing, Artificial

In recent years, many initiatives have been made to improve the quality and efficiency of healthcare services leading to the creation and standardization of health quality indicators in order to improve the interaction between clinicians, researchers, and healthcare managers, enabling the generation of evidence-based strategies. In this context, the rate of hospital readmissions is one of the most important indicators to measure the quality of healthcare services.

The use of quality indicators to measure healthcare performance across different hospitals has been considered of great value for the improvement of healthcare routines, enhancing the expenditure rationalization of health services. In this scenario, the adoption of the International Consortium for Health Outcomes Measurement (ICHOM) as a strategy to measure outcomes across hospitals has increased worldwide and it is now gaining familiarity among Brazilian hospitals.

Specifically in heart failure patients, the quality indicators can be measured by hard outcomes such as mortality, hospital readmissions or unscheduled visits to the emergency department. However, other measures should also be taken into account, especially patient-reported outcomes as quality of life, functional capacity, as well as the adherence to the treatment in terms of medications, diet and physical rehabilitation. In this sense, discharge instruction regarding the prescribed medications, dietary restrictions, regular physical activities, weight monitoring and the importance of follow-up appointments play a significant role in the promotion of successful treatment. Another factor of great impact for the success of the treatment is the close contact between the healthcare providers and the patient, by telephone or text messages, in order to reinforce the main instructions and to detect early signs of clinical decompensation. From this perspective, knowing the rate of unscheduled visits may be a good start for evaluating our results.

The study of Warpechowski Neto et al.^1^ showed a high incidence of unplanned visits after defibrillators (ICD) or cardiac resynchronization therapy (CRT) devices implantation. Device-related complications were seen in 7% of readmitted patients. On the other hand, in 24.6% of the patients, the reason for an unscheduled visit was cardiac and non-cardiac clinical conditions.

A 12-month follow-up study of 713 patients undergoing cardiac implantable electronic devices (CIED) procedures, published by Silva et al.^2^ in 2016 the Arquivos Brasileiros de Cardiologia, showed that the odds of new readmission in CRT patients was 1.6 times higher than in the general group of patients studied and that ICD implant increased this chance by 4.2 times. This study also showed that the mortality was 2.2 times higher in patients with left ventricular dysfunction and 2.3 times higher in those who used warfarin.^2^

In a prospective multicenter study conducted by the Instituto do Coração do Hospital das Clínicas da Faculdade de Medicina da Universidade de São Paulo, which included 3,550 patients from 9 geographically distributed cardiology centers, the 12-month readmission rate was 23.8%, 24.0% and 38.3% and the mortality rate was 9.4%, 11.5% and 18.3%, respectively, for ICD, CRT-P and CRT-D initial implantation. Device-related complications, heart failure decompensation and non-cardiac causes were the reason for readmissions in 15.5%, 22.1% and 19.2% of patients and for death in 3.7%, 15.7% and 51.8%, respectively.^3^

Analysis of the United States Nationwide Readmissions Database, which included 70,223 patients submitted to CIED implantation, showed a 30-day hospital readmission rate of 12%. Besides identifying several readmission predictors, mostly related to patient's comorbidities, these 30-day readmissions resulted in an additional median cost of US $30,692 per patient, which reinforces the importance of establishing strategies to reduce in-hospital readmissions.^4^

Analysis of the above-mentioned information shows the great importance of monitoring outcomes in CIED patients after the hospital discharge. In this sense, the conduction of prospective registries for all CIED types and procedures will provide us with more accurate information about the clinical practice in a real-world scenario. Ultimately, this knowledge will be critical for physicians, hospitals, and healthcare payers to know the results of their activities, in order to improve patient outcomes, while reducing costs. As important as knowing our results is to establish strategies to minimize complications. And certainly, reducing the rate of unscheduled visits should be a clear goal to be pursued.
